# Sensing Performance Analysis on Quartz Tuning Fork-Probe at the High Order Vibration Mode for Multi-Frequency Scanning Probe Microscopy

**DOI:** 10.3390/s18020336

**Published:** 2018-01-24

**Authors:** Xiaofei Zhang, Fengli Gao, Xide Li

**Affiliations:** 1 Department of Engineering Mechanics, AML, Tsinghua University, Beijing 100084, China; zhang-xf13@mails.tsinghua.edu.cn; 2 Center for Nano and Micro Mechanics, Tsinghua University, Beijing 100084, China; 3 Institute of Microelectronics of Chinese Academy of Sciences, Beijing 100029, China; gaofengli@gmail.com

**Keywords:** quartz tuning fork-probe, high mode, dynamic mechanical behavior, finite element method, multi-frequency scanning near-field optical microscopy

## Abstract

Multi-frequency scanning near-field optical microscopy, based on a quartz tuning fork-probe (QTF-p) sensor using the first two orders of in-plane bending symmetrical vibration modes, has recently been developed. This method can simultaneously achieve positional feedback (based on the 1st in-plane mode called the low mode) and detect near-field optically induced forces (based on the 2nd in-plane mode called the high mode). Particularly, the high mode sensing performance of the QTF-p is an important issue for characterizing the tip-sample interactions and achieving higher resolution microscopic imaging but the related researches are insufficient. Here, we investigate the vibration performance of QTF-p at high mode based on the experiment and finite element method. The frequency spectrum characteristics are obtained by our homemade laser Doppler vibrometer system. The effects of the properties of the connecting glue layer and the probe features on the dynamic response of the QTF-p sensor at the high mode are investigated for optimization design. Finally, compared with the low mode, an obvious improvement of quality factor, of almost 50%, is obtained at the high mode. Meanwhile, the QTF-p sensor has a high force sensing sensitivity and a large sensing range at the high mode, indicating a broad application prospect for force sensing.

## 1. Introduction

Quartz tuning forks (QTFs), where an optical fiber is attached to one of the prongs, are originally used as a distance control unit to monitor the tip-sample distance in scanning near-field optical microscopy (SNOM) [1,2,3]. Subsequently, the QTF with a probe attached on one of its prongs, called a quartz tuning fork-probe (QTF-p) [4], has been introduced into scanning probe microscopy (SPM) and operated in two typical working modes, the shear mode and vertical mode. The shear mode, where the probe vibration direction is parallel to the surface of the sample [5,6] and the vertical mode or the so-called qPlus configuration, where the probe vibration direction is perpendicular to the sample surface [7,8]. Compared with the traditional cantilever beam or cantilever combined light-lever type scanning probe microscope [9,10,11,12], the QTF-based SPM has the ability of self-actuating and self-sensing through the piezoelectric effect of the bending prong [13]. In particular, the high spring constant of the prong prevents the probe tip from jumping to the sample surface and allows the vibration amplitude of the tip to be maintained at a stable lower value to the nanometer scale. Currently, QTFs have been widely used for high-resolution microscopy [14,15], biosensors [16] and force sensors [17,18,19,20].

As the QTF-p has become increasingly used for the quantitative measurement of micro-forces and interactions, its dynamic response and sensing performance have attracted much attention. Previously, the dynamic behaviors of QTF at low mode are extensively studied. For example, with regard to the dynamic response of the QTF-p, Khaled et al. [21] used a single degree of freedom model to achieve viscous friction and elastic shear stress. Castellanos-Gomez et al. [22] presented two coupled oscillator models to investigate the coupling effect between the QTF prongs. Labardi et al. [23] investigated the dynamics of force microscope probes by simply modeling the QTF and probe system as two coupled damped harmonic oscillators. Gao and Li [24] established a coupling dynamic equation of a QTF-p sensor both at its longitudinal and lateral vibrations, where the QTF and probe were treated as deformable structures. The interaction between the probe and the sample surface, induced by the Van der Waals force and the viscosity of the liquid film, was investigated. About the sensing performance of QTF-p at low mode has also been investigated by experimental tests and the finite element method (FEM). Such as, Oria et al. [25,26] presented an electromechanically finite element model to analyze both the electrical and mechanical behaviors of the QTF and determined its static spring constant. Kim et al. [27] measured the effective stiffness of qPlus sensors and compared the results with those obtained from cantilever beam theory. Omur et al. [28,29] investigated the optimization of qPlus sensor assemblies based on FEM. Recently, Gao and Li [30] discussed the effects of various parameters on the resonance frequency and quality factor of the QTF-p by combining the experimental and FEM analyses. In short, much achievements on investigation of the dynamic behavior of the QTF-p at the low mode have been obtained.

A thorough review of methods of multifrequency scanning microscopy can be found in [31]. With the development of the multi-frequency scanning near-field optical microscopy (MF-SNOM) and its application to map near-field optically induced forces [32] and photoinduced force microscopy [33], the high mode dynamic characteristic becomes the key issue to obtain high accuracy measurement. However, the related researches on understanding the properties of amplitude-frequency and sensitivity of force sensing of the QTF-p at the high mode are insufficient. Particularly, the following questions are of interest to researchers: What is the dynamic behavior of the high mode of QTF-p? How do the connected glue and probe parameters influence the dynamic response of QTF-p? What are the differences between the high and low modes on the force sensing sensitivity?

In this paper, we investigate the vibration performance of QTF-p at high mode based on the experiment and FEM. The frequency spectrum characteristics are obtained by our homemade laser Doppler vibrometer system. The effects of the properties of the connecting glue layer (damping coefficient, Young’s modulus and adhesive thickness) and the probe features (diameter, extension length, configuration and material type) on the dynamic response of the QTF-p sensor at the high mode are investigated. We find that the material and geometrical properties of the glue layer and the probe have a significant influence on the sensing performance of QTF-p vibrating at the high mode. Despite the use of trace amounts of epoxy glue between the prong of the QTF and the probe in bottom probe connection configurations, the outside and inside connection configurations are more suitable to maintain the excellent dynamic performance of the QTF-p. The symmetrical configuration cannot improve the high sensing sensitivity because a greater amount of the glue has a substantial effect on the dynamic behavior of the QTF-p. Compared with the two modes, the quality factor (*Q*-factor) in the high mode is significantly better than that in the low mode. For the force sensing resolution, the QTF-p sensor has a high force sensing sensitivity and a large sensing range at the high mode.

## 2. Characterization of Geometric, Material and Frequency Spectrum Characteristics

**Figure 1:**
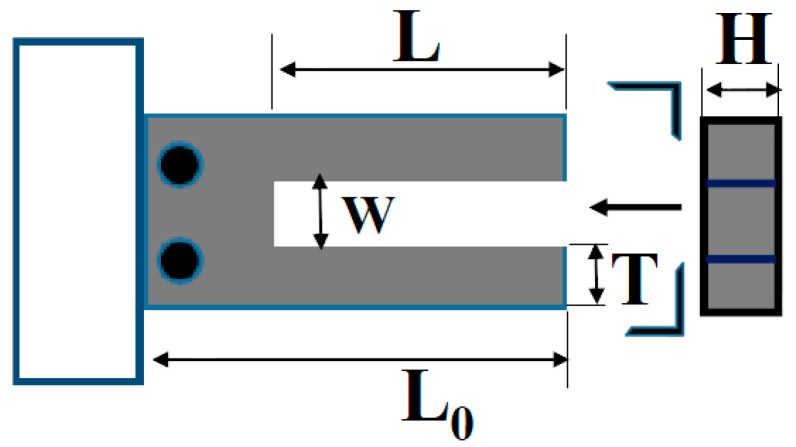
Schematic diagram of QTF.

A commercial bare QTF (JU-308, Suzhou Fengguang Electronics Co., Ltd, Suzhou, China) with a nominal resonance frequency of 32.768 kHz at the low mode was selected. The geometric parameters shown in Figure 1 obtained by the average of ten measurement results under an optical microscope (HIScope KH-3000, Hirox, Tokyo, Japan) were the prong thickness *T* = 0.606 ± 0.006 mm, gap of two prongs *W* = 0.266 ± 0.003 mm, total length *L*_0_ = 6.034 ± 0.023 mm and the prong length and height *L* = 3.783 ± 0.016 mm and *H* = 0.354 ± 0.004 mm, respectively. The main structures the QTF are made by the quartz and the attached electrode layer is chromium film.

The manufacturing error and microstructure difference of the material mean that the structure damping coefficient of the QTF is within a certain range, which leads to a different *Q*-factor. A tungsten wire and glass fiber probe are chosen as the probe to sense the force, while the prong and probe are connected by the epoxy glue. The material parameters of the QTF and the probe are provided in Table 1 along with references to previous work [28,30,34,35].

**Figure 2:**
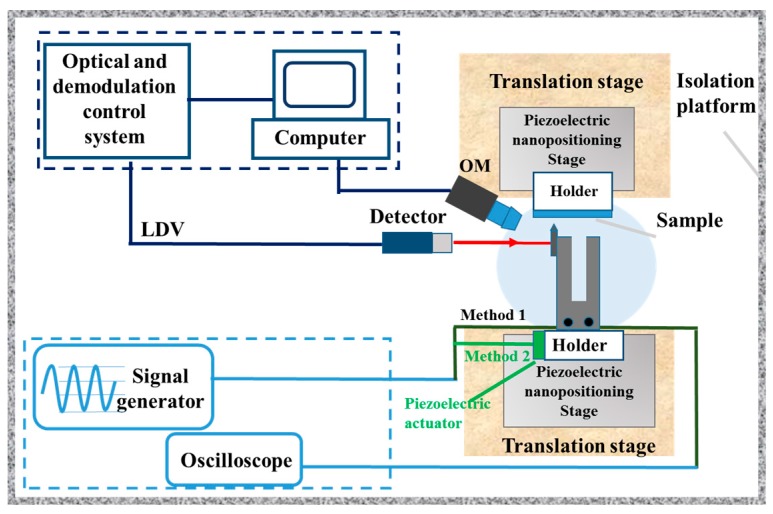
Schematic of the QTF testing system. Four parts are included: QTF-exciting unit, amplitude-frequency measurement unit, microscope and displacement control unit.

**Figure 3:**
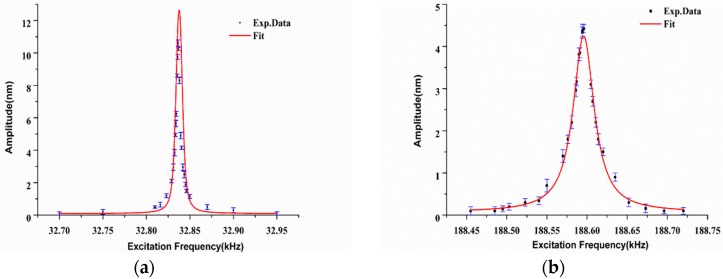
The typical experiment amplitude-frequency curve of the QTF lateral (shear) vibrating at the (**a**) low mode and (**b**) high mode.

To obtain the basic dynamic parameters (resonance frequency and *Q*-factor) of the QTF at high mode, we experimentally measured the amplitude-frequency curve based on the equipment in our laboratory, as shown in Figure 2. The system consisted of four parts: QTF-exciting unit, amplitude-frequency measurement unit, a microscope and a displacement control unit. The electrically driven mode (method 1 in Figure 2) or mechanically driven mode (method 2 in Figure 2, piezoelectric actuator (NAC2024, Harbin Core Tomorrow Science and Technology Co., Ltd, Harbin, China) was used as the mechanically exciting source) can be used to excite the sensor suitable for different working conditions. A signal generator (AFG-2225, Gwinstek, Taiwan, China) was used to provide a sweep frequency function for driving the sensor directly or the piezoelectric actuator. The homemade laser Doppler vibrometer system is a laser coherent detection system based on a Mach-Zehnder configuration. It can achieve non-contact amplitude measurement of QTF in real time utilizing Doppler effect when the laser spot is focused on the prong surface of the QTF. The system consists of four parts: a laser, an acousto-optic modulator (AOM), a detector and a demodulation control unit. The basic parameters of the system are: working distance: 5 cm or 20 cm; diameter of laser spot: 8 µm; displacement resolution: 50 pm; the range of frequency measurement: 0–200 kHz; sampling rate: 2 MS/s. The detailed information can be found in references [36,37]. The oscilloscope could display the reverse piezoelectric signal produced by the vibration of the QTF, which could then be used for the situation where the displacement or amplitude of the probe cannot be measured by the optical method. A piezoelectric nanopositioning stage (XP-363, XP-611, Harbin Core Tomorrow Science and Technology Co., LtdXMT, Harbin, China) provided precision translation with a displacement resolution of 0.6 nm under the close-loop control mode. An optical microscope (HIScope KH-3000, Hirox, Tokyo, Japan) with a long work distance objective lens was used to observe and then provide the positions of the probe and samples. In order to achieve the amplitude measurement of QTF, the implementation process is as follows: first, adjusting the distance between the detector of LDV and the surface of QTF prong to make the laser focus on the tested surface. Second, the QTF is excited with a sweeping frequency mode by a piezoelectric actuator and the amplitude of the prong is measured simultaneously by the LDV system in every frequency. Finally, the amplitude-frequency curve is obtained to extract resonance frequency and *Q*-factor. The typical amplitude-frequency curve of the used QTF are shown in Figure 3 with the resonance frequency of 32.837 kHz and the *Q*-factor of 10,946 for low mode and the resonance frequency of 188.562 kHz and the *Q*-factor of 11,080 for high mode. The resonance frequency and *Q*-factor of QTF used in the calculation were 32.8 ± 0.3 kHz and 11,000.0 ± 400.0 for low mode and 188.5 ± 0.4 kHz and 11,100.0 ± 600.0 for high mode respectively, the results from the average of measuring ten QTFs.

## 3. Optimization Design of QTF-p on Vibration Performance at the High Mode

### 3.1. Optimization Guidance on the Position of the Attached Probe

The high *Q*-factor of QTF-p is critical to provide high force sensing sensitivity and develop effective dynamic force sensor, meanwhile, the higher resonance frequencies give rise to larger frequency shifts in frequency modulation mode [28,30]. In order to obtain high-performance sensor for force sensing at high mode, the harmonic analyses based on FEM simulation were used to optimize design of the QTP-p sensor.

**Figure 4:**
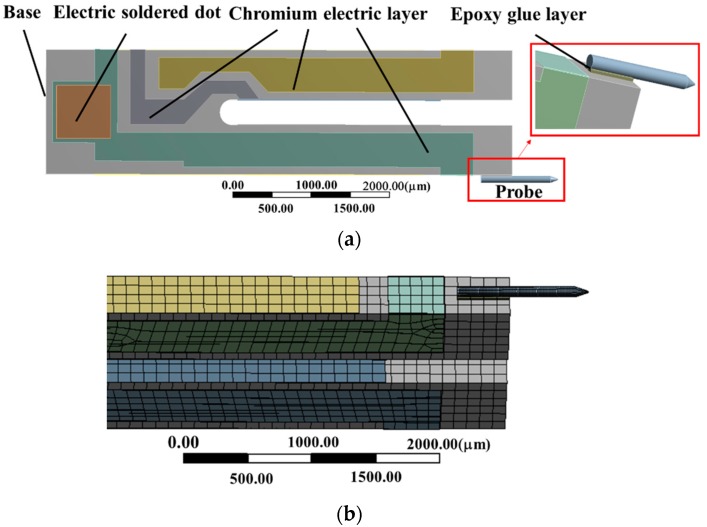
(**a**) QTF-probe and (**b**) meshing map in the numerical calculation.

**Figure 5:**
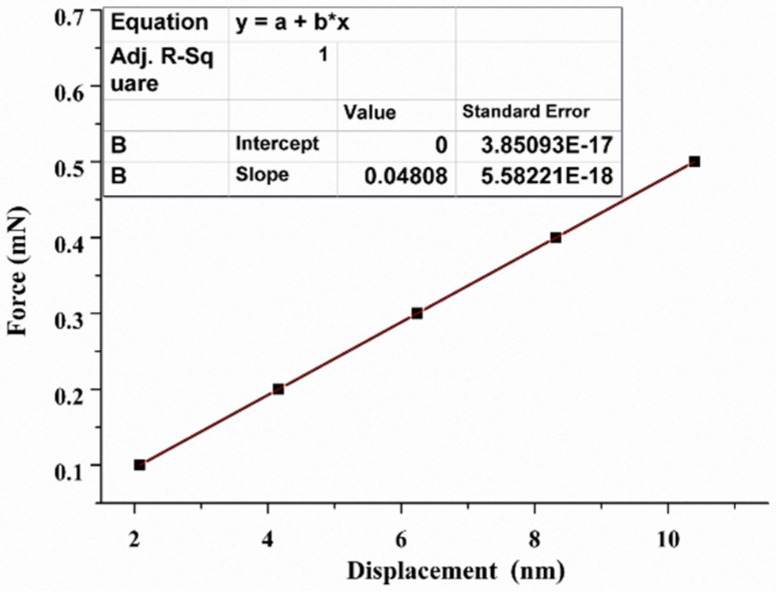
Displacement curve with an increasing force for calculating the spring constant *k* of a bare QTF.

The finite element model of QTF-p was established in Ansys Workbench environment and the geometric and material parameters are selected from Table 1. Owing to the small vibration amplitude of QTF, the linear elastic solid elements were used to model the body of the QTF and automatic grid meshing was employed. The grid meshing was fine enough to ensure the convergence of the calculated results. The base of the QTF (shown in Figure 4a) was adopted as the fixed boundary condition for simulating the actual condition. Two symmetrical bending moments were applied on the end of a prong as the excitation load. The oscillation amplitude of the prong depended on the value of the bending moment. It is worth noting that the electric pad and solder joints were modeled as the actual configuration on the QTF (shown in Figure 4a), which made the model more accurate. The simulation value of the resonance frequency for the QTF was 32.707 kHz at the low mode and 188.446 kHz at high mode with a 0.2% and 0.1% error compared to experiment results. Applying a constant force at the end of one prong, we were able to calculate the spring constant *k* through fitting of the force-displacement curve (Figure 5) which provided *k* = 48,077 N/m. Based on the geometrical calculation method [27], the spring constant *k* of the QTF can be expressed as $k=2\times 3.83{\left(E_{q}\rho_{q}{}^{3}\right)}^{1/4}\times H\times T^{3/2}\times f_{0}{}^{3/2}$, where *E_q_, ρ_q_* and *f*_0_ are the elastic modulus, density and resonance frequency of the low mode, respectively. Substituting the relevant parameters from Table 1 into the above formula, we obtained the value of the spring constant of 46,809 N/m, which was consistent with the FEM results. In the calculation, the value of the damping coefficient of quartz and chromium was chosen as 7 × 10^−6^ Ns/m. Owing to QTF oscillating in the atmosphere, additional viscous damping should be considered [38]. Therefore, the value of the constant damping ratio of QTF was selected as 3.9 × 10^−5^ by referencing the average experimental value of the *Q*-factor obtained in the harmonic response experiments in the atmosphere, which finally yielded the simulation values of the *Q*-factor 11,087 (low mode) and 11,193 (high mode), respectively. These calculated and experimental results showed the effectiveness of the numerical model.

**Figure 6:**
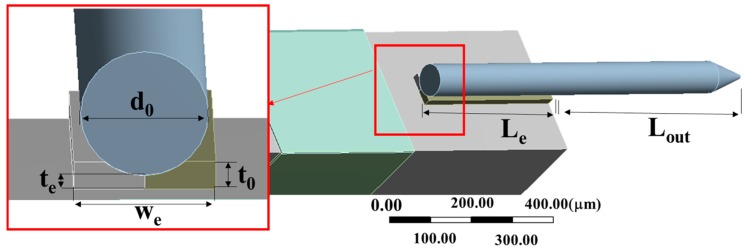
Model of the probe connection.

To explore the influence of the probe on the dynamic response of QTF-p at the high mode, the base configuration of the QTF-p is shown in Figure 4a. The linear elastic solid elements were used to model probe and epoxy glue layer. The probe was connected to one prong by the epoxy glue layer, while the shape of the glue was approximated to be rectangular based on the manufacturing method. The interfaces between the glue layer and prong, glue layer and probe were bonded. The mesh density was increased at the connection region between the probe and the prong in Figure 4b. The sketch of the probe connection is shown in Figure 6. The symbols *L_e_*, *W_e_* and *t_e_* represent the length, width and thickness of the glue layer, respectively. The probe diameter expressed as *d*_0_ represents the diameter of the cylindrical part of the probe. Owing to the attachment of the probe, an immersion phenomenon will occur, while the immersion depth is represented by the total height *t*_0_ subtracting the glue height *t_e_*.

The effect of the attached probe multi-parameter on the sensing performance of the QTF-p working at the high mode was investigated in this section. These parameters were the material type, geometrical dimension and the configuration of the probe connection.

**Figure 7:**
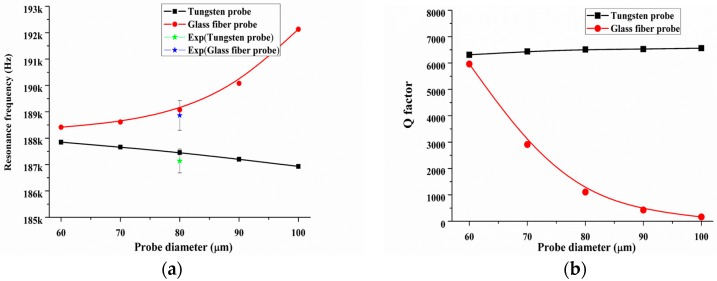
(**a**) Resonance frequency and (**b**) *Q*-factor changing with the increase of the probe diameter for tungsten and the glass fiber probes. The five-pointed star symbols denote experimental results.

First, we explored influence of two types of material on the resonance frequency and *Q*-factor. Here, the damping coefficient, elastic modulus and thickness of the epoxy layer were set to 0.2 Ns/m, 2 GPa and 10 µm, respectively. *L_e_* and *w_e_* were set as 500 µm and 100 µm, the immersion height *t_e_* was maintained at 10 µm, a tungsten wire or a glass fiber probe have been most frequently used in the previous work [4,6,29,32]. First, the calculation results on the changing of resonance frequency and *Q*-factor of tungsten-based and glass fiber-based QTF-p with the increase of the probe diameter are shown in Figure 7. The total probe length was chosen as 1 mm, extension length *L_out_* was 500 µm, the radius of the probe tip was 5 µm and its cone angle was 30°. The diameter was chosen from 60 µm to 100 µm and these parameter settings were suitable for the actual conditions and have been most used elsewhere [20,29,39]. We found that the *Q*-factor decreased rapidly with the increase of the diameter of the glass fiber probe. The value of the *Q*-factor almost decreased by 97% with the change of the diameter of glass fiber from 60 µm to 100 µm. In contrast, the *Q*-factor exhibited a small increase of approximately 4% for the tungsten probe. The main reason was that the extension part (‘free’ part) of probe had its own eigenmodes acting as a deformable beam ‘fixed’ at one end of the prong, while its own vibration performance had a big effect on the dynamic behavior of QTF-p. If a certain order eigenfrequency of probe was regulated to close to the resonance frequency of the QTF-p, the larger disturbance of the probe will appear to increase the structural damping of the QTF-p resulting in the rapidly decreasing of *Q*-factor. In contrast, the *Q*-factor can be improved when regulated eigenfrequency of probe was far away the resonance frequency of QTF-p. In our case for tungsten wire, the fundamental eigenfrequency of tungsten probe (the eigenfrequency value in diameter 60 µm was larger than the value of the resonance frequency of the QTF-p by our calculation) was gradually away from the resonance frequency of QTF-p with the increase of probe diameter. This leaded to the stability reinforcement of QTF-p and caused the *Q*-factor increasing. For glass fiber probe, its stiffness smaller than that of the tungsten probe. This made a higher mode be turned into a primary mode to disturb the dynamic characteristic of QTF-p. This higher eigenfrequency of glass fiber probe (the eigenfrequency value was smaller than the value of the resonance frequency of the QTF-p) was gradually got closer to the resonance frequency of QTF-p with the increase of the probe diameter from 60 µm to 100 µm. Therefore, the disturbance from the probe was significant and the *Q*-factor of QTF-p decreased rapidly.

**Figure 8:**
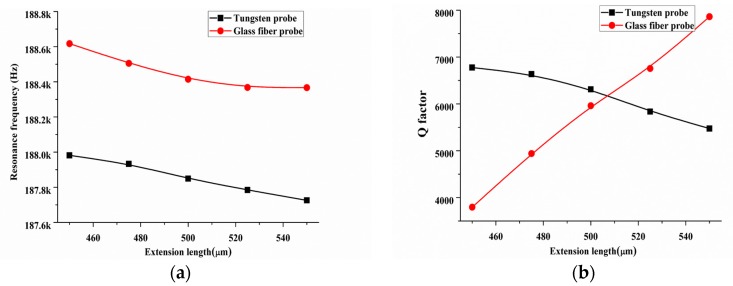
(**a**) Resonance frequency and (**b**) *Q*-factor changing with the increase of the extension length for tungsten and the glass fiber probes.

Meanwhile, we explored the influence of the extension length *L_out_* (shown in Figure 6) on the resonance frequency and *Q*-factor. The extension length was chosen from 450 µm to 550 µm and the probe diameter was set 60 µm. The calculation results on the changing of resonance frequency and *Q*-factor with the increase of the extension length for tungsten and the glass fiber probes were shown in Figure 8. The *Q*-factor of tungsten-based QTF-p decreased by 19.2%. On the contrary, the *Q*-factor of glass fiber-based QTF-p increased by 107.2% from 450 µm to 550 µm. For tungsten wire, the eigenfrequency of tungsten probe gradually got closer to the resonance frequency of QTF-p with the increase of probe extension length from 450 µm to 550 µm, which weakened the stability of QTF-p and caused *Q*-factor decreasing. For glass fiber, a higher eigenfrequency of glass fiber probe was gradually away from the resonance frequency of QTF-p with the increase of probe extension length from 450 µm to 550 µm. So, the disturbance from the probe was reduced resulting in the increasing of *Q*-factor of QTF-p. In conclusion, the vibration performance of ‘free’ part of probe played a decisive factor to obtain a higher *Q*-factor QTF-p sensor. The parameters of extension length, diameter and material type of the probe should be suitable chosen to ensure a high *Q*-factor sensor. Particularly, the extension length was an important parameter that can be easily tuned as part of the fabrication process more than diameter.

**Figure 9:**
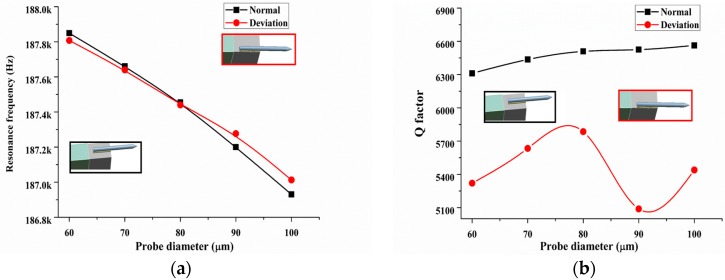
(**a**) Resonance frequency and (**b**) *Q*-factor changes with an increase of the tungsten probe diameters under a deviation of the probe position.

Second, for ease of preparation and handing, sometimes the probe is not exactly at the side center of the prong. The effect of the position deviation of the probe at the high mode vibration analyzed by FEM is shown in Figure 9. The maximum position deviation of the probe, from the side face center to its edge, was calculated. The high mode resonance frequencies of the QTF-p were almost the same with the change in diameter shown in Figure 9a for the two positions. The reason is that the stiffness and mass of the system are not changed under this condition. However, the *Q*-factor shows a larger difference with a change in the probe diameter. This result indicates that the position deviation changes the structural symmetry of the QTF-p and leads to an increase of the structural damping and decrease of the *Q*-factor. It also causes the *Q*-factors to change irregularly with the probe diameter. Therefore, the symmetrical structure is an important factor that affects the high frequency sensing performance of the QTF-p sensor.

**Figure 10:**
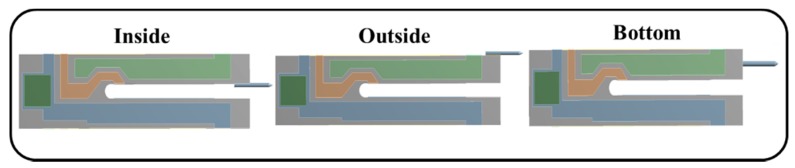
Three kinds of QTF-p configurations.

**Figure 11:**
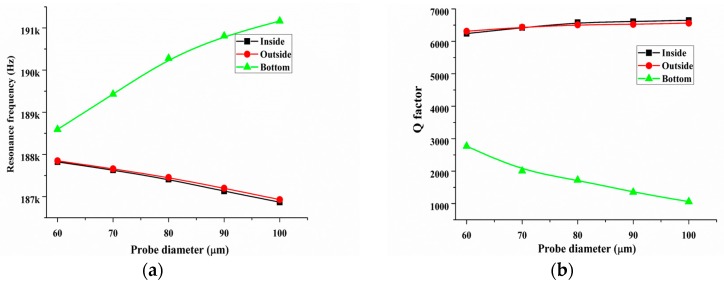
(**a**) Resonance frequency and (**b**) *Q*-factor with the increase of the tungsten probe diameter under the probe connection configurations of inside, outside and bottom.

Next, although an outside connection of the probe is widely used [8,18,32], the configurations of the probe inside and the end connected are also used in the application of the QTF-p sensor for different working conditions [17]. In this section, we compare the effects of three kinds of configurations (Figure 10) based on FEM at the high mode and provide some parameters for optimizing design of QTF-p configurations. Figure 11 shows almost the same influences on the dynamic response for the configurations of the inside and outside probe assemblies and the difference for the bottom probe assembly, where such a connection configuration reinforced the structural stiffness and increased the structural damping. Although it is beneficial to maintain the structural symmetry under the bottom type, the structural damping increased. Meanwhile, the bonding strength was reduced in this configuration because the adhesive area was reduced to the size of the cross-sectional area of the probe. Therefore, considering the assembly and practical applications, the outside assembly configuration was the preferred configuration.

**Figure 12:**
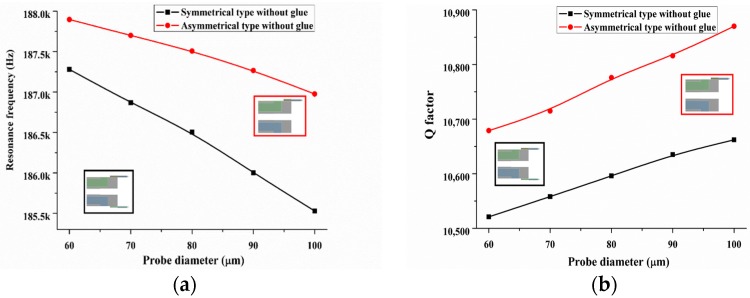

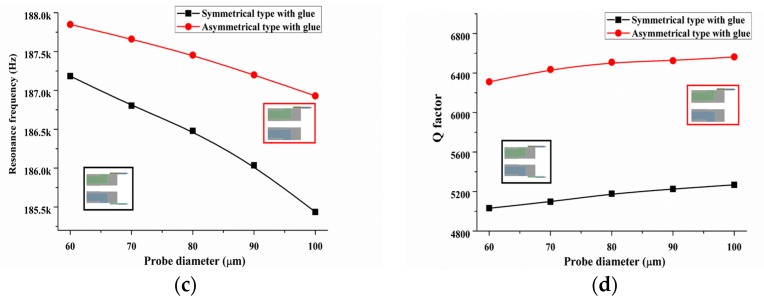
Resonance frequency and *Q*-factor of QTF-p without glue (**a**,**b**) and with glue (**c**,**d**) accompanying the increase of the tungsten probe diameters for two typical probe configurations (asymmetrical and symmetrical).

Last, the bare quartz tuning fork possesses excellent dynamic properties owing to its symmetrical structure. Although the probe size is small, its introduction including the glue layer undoubtedly changes the structural symmetrical balance. The breaking symmetrical balance is also a key factor that decreasing sensor’s *Q*-factor. Thus, it is important to known what factors (breaking of symmetrical balance or additional probe) is primary on the decreasing of *Q*-factor. In order to keep the structural symmetry of QTF-p sensor, two symmetrical probes were assembled to the outside of the prongs of the QTF, as illustrated in Figure 12. For comparison, one probe with the outside assembly is also shown in Figure 12. First, to eliminate the effect of additional glue on the calculation result, we only discuss the case where the probe was connected with the prong without glue. The FEM calculated results of the resonance frequency and *Q*-factor with the changes of the diameter of the probe are shown in Figure 12a,b, respectively. We observed that although the symmetrical configuration made the structure balanced, the value of the *Q*-factor in the symmetrical configuration was smaller than that in the asymmetrical configuration. This result indicated that the increasing of the structural damping owing to the breaking of symmetrical balance was less than that of the self-damping of the probe introduced to the QTF-p structure. Follow, we discuss the actual situation where the probe was connected to the prong with a glue layer, where the parameters of the glue layer were the same as in the first part of this section. The calculated results showed the same trend on the resonance frequency and the *Q*-factor for both types. The changes in the value of these parameters for the asymmetrical configuration were smaller than those for the symmetrical configuration. For example, the resonance frequencies of the asymmetrical and symmetrical configurations were reduced by approximately 0.5% and 1%, respectively, when the probe diameter was increased from 60 µm to 100 µm. Similarly, the *Q*-factor was increased by 4% in the asymmetrical configuration and 4.7% for the symmetrical case. Meanwhile, we found that the *Q*-factor of the symmetrical type was decreased by approximately 20% compared with that of the asymmetrical type. The above results showed that the competition of the symmetry breaking and damping characteristics determine the final dynamic performance of the QTF-p sensor and probe introduction including the glue layer is major factor decreasing the *Q*-factor. So, symmetrical probes configurations should not be chosen.

The main calculation error came from the selection of geometric parameters of QTF-p can be neglected. As a typical example, based on the calculation on the resonance frequency changing with the increase of the probe diameter for tungsten probe, we found that error has a little influence on the analyses of overall curve trend. Due to the overall curve trend that is mainly considered in our paper, the error bars of other figures were not shown.

### 3.2. Optimization Guidance on the Choosing of Epoxy Glue

The effect of the epoxy layer multi-parameter on the sensing performance of the QTF-p working at the high mode was investigated in this section, including the epoxy layer thickness, damping coefficient and Young’s modulus.

**Figure 13:**
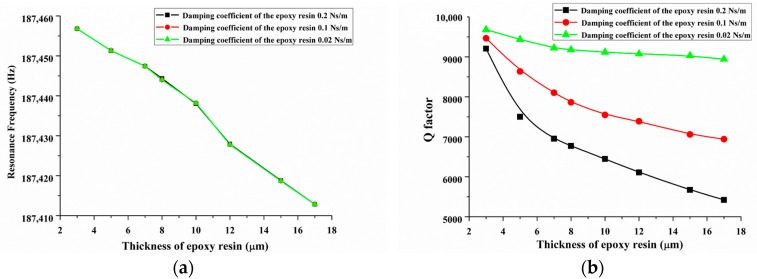
Relationship between the high mode (**a**) resonance frequency and (**b**) *Q*-factor with the thickness of the epoxy layer under three typical damping coefficient values.

First, we discuss the effect of the glue layer thickness on the resonance frequency and *Q*-factor of the QTF-p at the high mode under the condition of different glue layer damping coefficients. Figure 13 shows the effect of the glue thickness on the resonance frequency and *Q*-factor based on FEM calculation the high mode with three typical damping coefficients of the epoxy resin (0.2 Ns/m, 0.1 Ns/m and 0.02 Ns/m), where the elastic modulus of the epoxy resin was set to 2 GPa. The material of the probe was tungsten and its diameter and length were chosen as 80 µm and 1 mm, respectively. From Figure 13a, we observed that the resonance frequency decreased by 0.02% with the increase of the glue thickness (additional mass) from 2 µm to 17 µm, which indicates that the effect of thickness on the resonance frequency is negligible. Meanwhile, the resonance frequencies were almost equal to each other at the same thickness of the glue layer and independent of the glue damping coefficients. This result indicated that the damping coefficient of the epoxy layer had little influence on the resonance frequency of the QTF-p. In addition, the large influence on the *Q*-factor is shown in Figure 13b, which is caused by the damping coefficient and the thickness of the epoxy layer. The *Q*-factor of the QTF-p decreased rapidly with the increase in the thickness and damping of the adhesive glue layer. For example, the *Q*-factor fell 7.7% with the glue thickness changing from 2 µm to 17 µm when the damping coefficient was 0.02 Ns/m. As a comparison, the *Q*-factor fell 26.7% when the damping coefficient was set to 0.2 Ns/m. Therefore, the damping coefficient and thickness are the key factors to obtain a high quality QTF-p sensor for the force or the interaction sensing working at the high mode. So, the adhesive glue with a lower damping coefficient should be selected and a small amount of the glue should be used for the connection.

**Figure 14:**
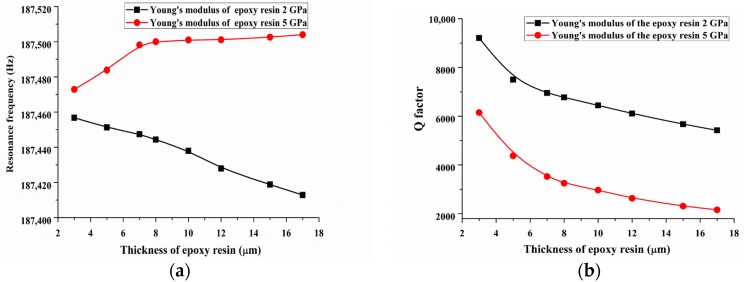
(**a**) Resonance frequency and (**b**) *Q*-factor with an increase of the Young’s modulus and thicknesses of the epoxy layer at the high mode.

Second, we discuss the influence of Young’s modulus of the epoxy layer on the dynamic response at the high mode. Figure 14 shows the FEM calculation result of Young’s modulus setting to 2 GPa and 5 GPa and the damping coefficient of the glue was set to 0.2 Ns/m. A high mode resonance frequency showed a different tendency when the elastic modulus of the epoxy layer was changed from 2 GPa to 5 GPa. In fact, the high mode resonance frequency Ω can be expressed as $\Omega \propto \sqrt{\frac{K+\Delta K}{M+\Delta M}}$, where *K* and *M* are the equivalent stiffness and mass of the QTF-p and Δ*K* and Δ*M* represent the changes of equivalent stiffness and the mass of QTF-p arising from the contribution of the changing Young’s modulus. The increase in the Young’s modulus will result in an increased stiffness of the epoxy layer. It is clear that the trend of the resonance frequency in Figure 14a is the competitive result of the increase of the system stiffness and the increase of the mass. But, the resonance frequency largest increased only 0.05% in our situation and the effect can be ignored. For the *Q*-factor of the QTF-p, relatively lower values were obtained for the higher modulus. For example, the *Q*-factor decreased by almost 33.2% when the Young’s modulus was changed from 2 GPa to 5 GPa when the glue layer thickness was set to 3 µm. This result indicates that the higher modulus value will strengthen the structural damping. Therefore, the relatively low modulus epoxy should be chosen for improving the force sensing sensitivity for high mode.

Owning to the grid meshing was fine enough to ensure the convergence of the calculated results, the calculation error on the resonance frequency and *Q*-factor can be ignored based on our calculation. The possible error comes from the accuracy of data extraction of frequency originally form the amplitude-frequency curve and the value of *Q*-factor. Our calculations show that the calculation errors caused by this factor has the maximum values of 0.05% and 0.1% for the resonance frequency and the *Q*-factor, respectively.

## 4. Discussion

### 4.1. Comparison of Force Sensing Ability of the QTF-p Sensor at Low and High Mode

**Figure 15:**
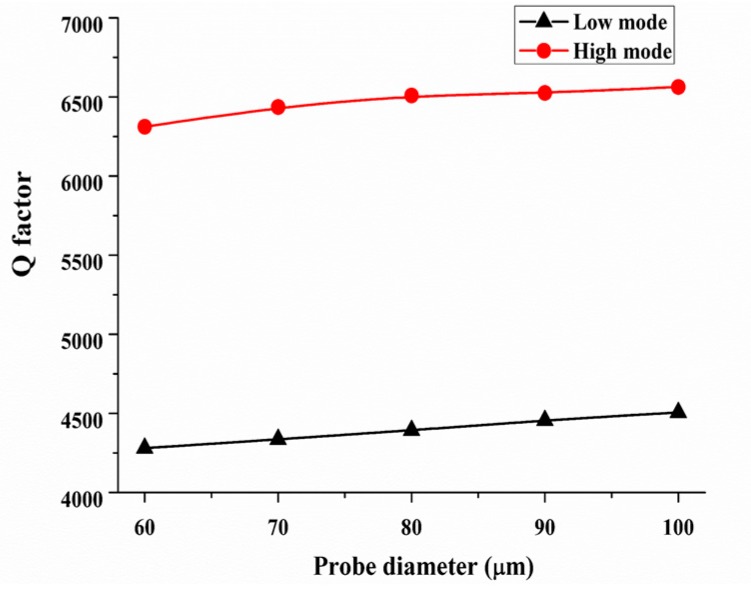
*Q*-factor of the QTF-p with changes of the tungsten probe diameter at the high and low modes.

We explored the force sensing differences of the QTF-p at the low and high modes. First, the effect of the probe on the *Q*-factor calculated by FEM for the two modes are shown in Figure 15. It is worth noting that *Q*-factors were reduced by 41.5% and 60.4% compared with the bare QTF for the high and low mode, respectively, when the probe diameter was 80 µm. This result shows that the damping property of the QTF-p sensor was less affected by the probe diameter at the high mode.

**Figure 16:**
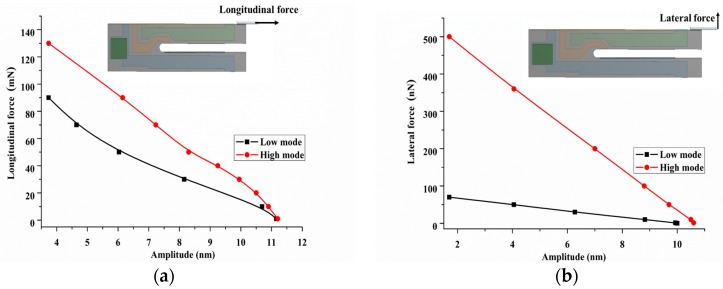
Resonance amplitude of the QTF-p under (**a**) the longitudinal and (**b**) lateral interactions at two modes.

To investigate the force sensing ability of the QTF-p at the low and high modes, we calculated the force sensing range and sensitivity under longitudinal and lateral interactions being exerted at the probe tip. The QTF-p may undergo the two types of loads on probe tip (lateral force and longitudinal force), in which that the meaning of “lateral” and “longitudinal” is defined referencing the axial of prong of QTF-p (refer to Figure 16a,b). Both of two types force can change the dynamics of QTF-p, such as probe amplitude and resonance frequency. For the longitudinal interaction condition, the amplitude of the probe tip was set to 11.2 nm (this value of the amplitude is commonly used in force sensing) under the free vibration for the two modes. Figure 16a shows the change of the probe tip amplitude under the action of the longitudinal force. The amplitude of the probe tip was reduced to 3.7 nm when the longitudinal force was increased to 90 mN for the low mode and the longitudinal force was increased to 130 mN for the high mode. It indicates that a large range of force sensing can be achieved at the high mode for longitudinal force sensing. Similarly, the dynamic characteristic of the QTF-p was calculated under the lateral force loading and the results are shown in Figure 16b, where we selected the viscous interaction as the lateral force, which is proportional to the velocity of the probe moving along the lateral direction. The linear relationship between the lateral force and amplitude is shown for both modes. Examples of the relationship are the amplitude of probe tip decreased from 10 nm to 1.7 nm which led to the increase of the lateral force from zero to 70 nN at the low mode and the tip amplitude decreased to 1.7 nm to make the lateral force increase from zero to 500 nN at the high mode. This corresponded to a force sensing sensitivity of 8.4 nN/nm for the low mode and 59.7 nN/nm for the high mode (refer to Figure 16b). Obviously, with regard to the lateral force sensing, a higher force sensing sensitivity was achieved at the high mode. With regard to the force sensing resolution, if the measurement accuracy of the amplitude of QTF-p can reach 1 nm, the resolution of the force sensing would be 8.4 nN and 59.7 nN, respectively. These results indicate that the QTF-p sensor has a high force sensing sensitivity and a large range of force sensing at the high mode, as well as a high accuracy measurement and a small range of force sensing at the low mode. The difference of sensing range and sensitivity on lateral force (nN order of magnitude) and on longitudinal force (mN order of magnitude) were found owning to lateral stiffness is far smaller than longitudinal stiffness of QTF. In the micro force sensing field, the lateral force sensing plays a decisive role.

### 4.2. Comparison between Theory and the FEM Model of the Dynamic Behavior of the QTF-p at the High Mode

**Figure 17:**
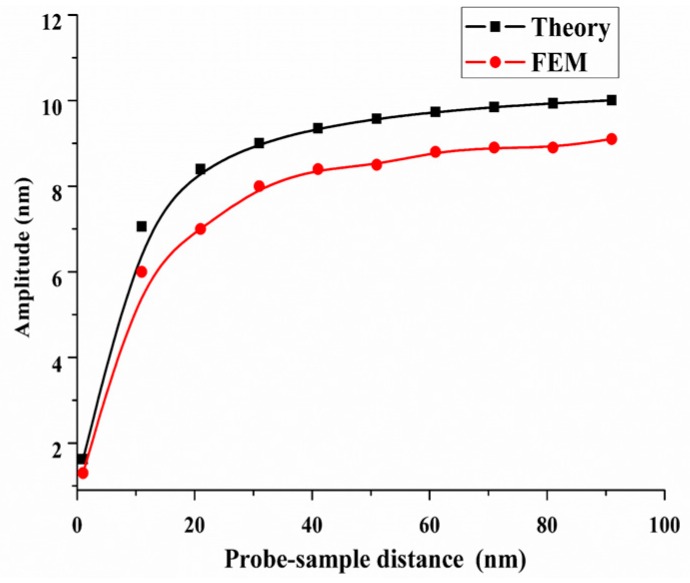
Approach curves of the QTF-p under a viscous resistance interaction.

In Section 3.1, we observed that the deformation of the probe had a larger influence at the high mode than at the low mode. In our previous work, we established a dynamic equation for the QTF-p under the lateral force with a deformable probe [24]. The control equation using linear vibration theory adopted to model on prong of QTF-p can be expressed as:

(1) $$Mq_{tt}\left(t\right)+Kq\left(t\right)=Cq_{t}\left(t\right)+F\cos\left(\Omega t\right)$$

where *q*(*t*) is lateral modal response. The *M*, *K*, *C*, *F* are the principal mass, stiffness, damping and equal exciting force. These parameters can be calculated utilizing the vibration mode shape and other parameters, including geometric dimension, material and dynamic parameters. In this condition, we discuss the dynamic response of the QTF-p under viscous resistance of a water film at the high mode and second symmetrical vibration mode shape was used in theory calculation. The viscous resistance exerted on the probe is $F_{d}=\gamma \nu A_{p}/d$, where γ is the coefficient of the viscosity of water is, *A_p_* is the contact area of probe, *d* is the distance between the tip and the sample and *v* is the velocity of the probe tip and the value of *C* is modulated by the viscous resistance. The corresponding parameters and solution process of the Equation (1) can be obtained in reference [24]. The Figure 17 shows the comparison of the approaching curves under a viscous resistance interaction obtained by a numerical calculation and theory analyses. In general, these results were coincident. The reason on the smaller values of the numerical results is that the influences of the electrode and the adhesive layer were taken into account during the calculation.

## 5. Conclusions

In this paper, we investigate the vibration performance of QTF-p at high mode based on the experiment and FEM. The frequency spectrum characteristics are obtained by our homemade laser Doppler vibrometer system. The effects of the properties of the connecting epoxy glue layer (damping coefficient, Young’s modulus and adhesive thickness) and the probe features (diameter, extension length, configuration and material type) on the dynamic response of the QTF-p sensor at the high mode are investigated. The results indicate the *Q*-factor decreases rapidly with the increase of the thickness and the damping of the glue layer. Moreover, the higher modulus value of the glue layer will strengthen the structural damping. A small deflection of tungsten probe during the vibration is achieved, which is beneficial to maintain the structural stability. The factor of extension length and diameter should be overall consideration for obtaining a high-quality sensor. Particularly, the extension length is an important parameter that can be easily tuned as part of the fabrication process more than diameter. Although it is beneficial to maintain the structural symmetry with the bottom probe connection configuration, the outside assembly configuration is the preferred configuration considering easy assembly and practical applications. For the practical use of the QTF-p sensor, the symmetrical configuration cannot always improve the *Q*-factor because the attached probe or a larger introduced glue layer will provide an additional damping effect to counteract the contribution of the symmetrical configuration. Compared with the low mode, the high mode has an obvious improvement on the quality factor of approximately 50%. The QTF-p sensor has a high force sensing sensitivity and a large sensing range at the high mode, indicating a broad application prospect for force sensing. Although the conclusion of this paper is based on the type of JU-308 QTFs. It is believed that these conclusions will also help us to understand the higher order performance of other types of QTFs.

## Figures and Tables

**Table 1:** Material parameters of the QTF and probe.

Material Parameters	Quartz	Chromium	Tungsten	Epoxy
Density (kg/m^3^)	2650	7190	1925	2
Young’s modulus (GPa)	78.7	279	411	2–20
Damping coefficient (Ns/m)	0.07–2 × 10^−4^	7 × 10^−6^	0.005	0.005–0.5
